# Thoracic Anesthesia and Cross Field Ventilation for Tracheobronchial Injuries: A Challenge for Anesthesiologists

**DOI:** 10.1155/2014/972762

**Published:** 2014-01-12

**Authors:** Sankalp Sehgal, Joshua C. Chance, Matthew A. Steliga

**Affiliations:** ^1^Department of Anesthesiology and Pain Medicine, University of Arkansas for Medical Sciences, Little Rock, AR 72205, USA; ^2^Department of Cardiothoracic Surgery, University of Arkansas for Medical Sciences, Little Rock, AR 72205, USA

## Abstract

Tracheobronchial injuries are rare but life threatening sequel of blunt chest trauma. Due to the difficult nature of these injuries and the demanding attributes of the involved surgery, the anesthesiologist faces tough challenges while securing the airway, controlling oxygenation, undertaking one-lung ventilation, maintaining anesthesia during tracheal reconstruction, and gaining adequate postoperative pain control. Amongst the few techniques that can be used with tracheobronchial injuries, cross field ventilation is a remotely described and rarely used technique, especially in injuries around the carina. We effectively applied cross field ventilation in both our cases and the outcome was excellent.

## 1. Introduction

Tracheobronchial injuries (TBI) are life threatening complications encountered in blunt chest and neck trauma. Tracheal injuries should be suspected in all patients involved in high speed motor vehicle accidents. The first successful repair of a bronchial rupture caused by blunt chest trauma was reported in 1947 by Kinsella and Johnsrud [1]. They are found in 0.8% of blunt thoracic trauma victims presenting for emergency surgery [2]. Tragically, 30% to 80% of these patients die before reaching the hospital [3].

Surgical repair remains the treatment of choice for such injuries. Due to the difficult nature of these surgeries, the anesthesiologist faces tough challenges securing the airway, controlling oxygenation and ventilation, undertaking one-lung ventilation, maintaining anesthesia during tracheal reconstruction with loss of ventilation to the atmosphere, and gaining adequate postoperative pain control. For appropriate management of these injuries in the operating room, modified anesthetic techniques and effective communication with the thoracic surgeon are important. Key considerations are avoidance of excessive preoperative sedation, maintaining spontaneous ventilation during intubation, using bronchoscopy to visualize and secure the airway, avoiding blind instrumentation [4, 5], single-lumen tube endobronchial intubation, cross field ventilation, and adequate postoperative pain control. Using these techniques, the outcome of our cases was excellent.

In the current case scenario, we shall discuss anesthetic management of two patients who presented to our level-1 trauma center, between years 2010 and 2012, with tracheobronchial injuries following severe blunt chest trauma in motor vehicle accidents.

## 2. Case Report Number 1

**Figure 1:**
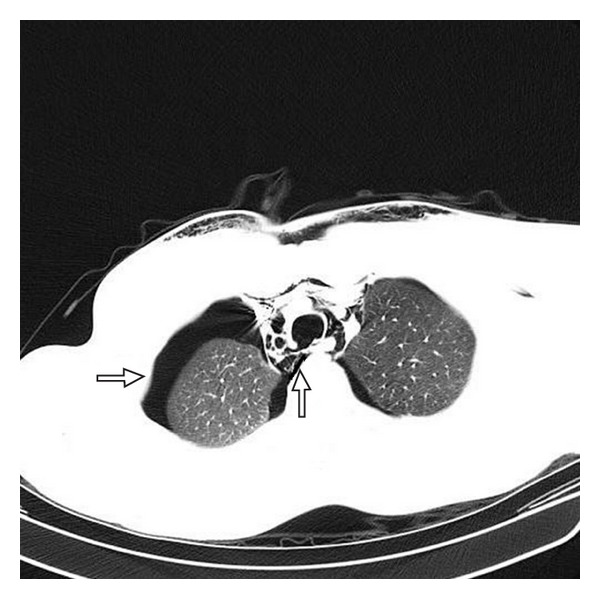
CT chest image depicting tear in the posterior part of the trachea with a right-sided pneumothorax.

A 20-year-old male presented following a motor vehicle accident. His physical findings included a right-sided tension pneumothorax which was treated with a chest tube, subcutaneous emphysema, and left clavicle fracture. CT of the thoracic spine revealed a longitudinal defect in the posterior membranous wall of his trachea extending into the carina (Figure 1). With an impending respiratory failure and the possibility of mediastinitis and subsequent sepsis with nonoperative management, it was decided to operate upon the tracheal tear. His past medical history included reflux disease, smoking, and alcohol consumption but no major cardiopulmonary problems. His vital signs were stable so far. The airway was anesthetized in preparation for an awake oral fiberoptic bronchoscopy and a radial arterial line was placed preoperatively. He was then transferred to the operating room and midazolam was titrated for sedation and anxiolysis. With the patient still awake, flexible bronchoscopy was performed to evaluate the posterior pharynx and arytenoids and to remove blood and debris from the airway. The bronchoscope was advanced into the left main-stem bronchus and a reinforced 7.5 size endotracheal tube was placed. The intubation was followed by induction using propofol, fentanyl, and cisatracurium and he was then placed on the ventilator thus ensuing one-lung ventilation via selective bronchial intubation. Due to the short length of the reinforced tube, it was replaced with a regular 7.5 size tube using a tube exchanger. He was then maintained under general anesthesia using sevoflurane with end-expiratory concentration of approximately 2%. Ventilator parameters included a tidal volume of 500 mL (approximately 7 mL/kg), a 12–14 cycles/min respiratory rate, and inspired oxygen fraction 0.55–0.7% and his oxygen saturation was maintained between 96 and 99%.

**Figure 2:**
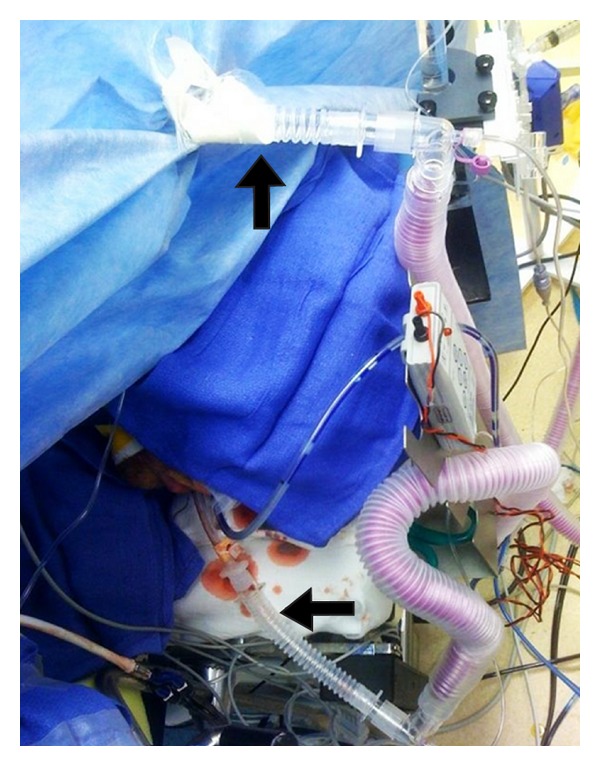
An oral endotracheal tube and a second cross field ventilation circuit that is passing through the drapes.

**Figure 3:**
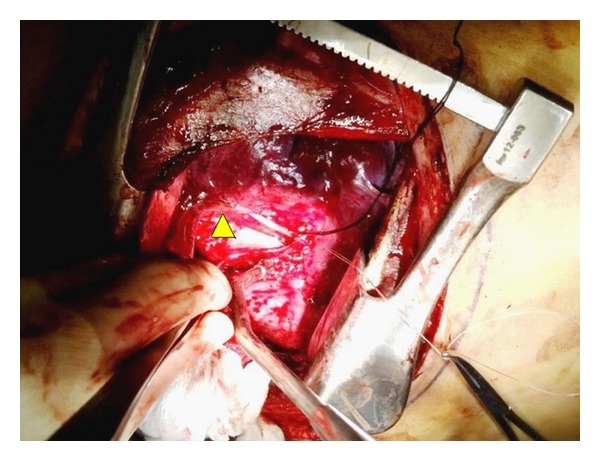
Cross field selective one-lung ventilation with a single-lumen endobronchial tube via the thoracotomy.

During the surgery, while briefly holding respirations, the endotracheal tube was withdrawn and a bronchoscope was used to visualize and assess the extent of the tear. A 9 to 10 cm long longitudinal tear was seen on the posterior membranous portion of the trachea. The tube was replaced into left main bronchus and selective left lung ventilation was undertaken. The patient was positioned in left lateral decubitus position. A right thoracotomy was done and the tracheal tear was noticed to extend from the right main-stem bronchus up to the apex of the chest. The cuff of the endotracheal tube was noted to be in the area requiring repair and hence cross field ventilation was planned. A size 6.0 sterile endotracheal tube was brought into the surgical field through the drapes, which was placed by the surgeon into the left main-stem bronchus via the thoracotomy. At the same time the oral endotracheal tube was withdrawn (Figure 2). Again selective left lung ventilation was undertaken. The endobronchial intubation via thoracotomy and cross field ventilation greatly facilitated the tracheal repair around the carina (Figure 3). A bronchoscopy using the fiberoptic scope was then performed through the oral tube and the tracheal repair was noted. Once the carina was almost completely repaired and sutures around it were ready to be tied, the cross field tube was withdrawn and the oral endotracheal tube was reinserted into the left main-stem bronchus over the fiberoptic bronchoscope. Now the remaining part of the trachea was repaired and the repair was reinforced with an intercostal muscle flap. This was followed by closure of the chest. Two right-sided chest tubes were then placed by the surgeon. Hemodynamics and electrocardiogram remained stable. The patient's paralysis was fully reversed and he was extubated in the operating room and transferred to the intensive care unit.

He had an uneventful postoperative course and was discharged on tenth postoperative day.

## 3. Case Report Number 2

A 26-year-old male presented following a rollover motor vehicle accident. He was an unrestrained driver and was hemodynamically stable. He did not have any open injuries. He had a past medical history of asthma, drug and alcohol use, and smoking. Upon physical examination he had left-sided chest tenderness, bilateral breath sounds which were slightly decreased on the left side, subcutaneous emphysema in the neck, and normal heart sounds. He had a C-collar in place and hence poor mouth opening and difficult airway examination. Chest X-ray revealed bilateral pneumothoraces and left 1st and 3rd rib fractures. A CT of the thoracic spine confirmed bilateral pneumothoraces and showed left pulmonary contusion and extensive air tracking along soft tissue planes of the neck with a defect of posterior trachea at approximately T9 level; the latter finding was suspicious for tracheal injury. For the blunt tracheal rupture and bilateral pneumothoraces, a bronchoscopy, right thoracotomy, and tracheal repair with intercostal muscle flap was planned.

The patient was taken to the operating room. Due to the presence of a hemopneumothorax on the left, it was decided to operate on the right side. After induction with fentanyl, propofol, and succinylcholine, a fiberoptic bronchoscopy was performed to evaluate the airway and a several centimeters long longitudinal split was noted along the entire posterior wall of the trachea that extended to the carina. There was also a significant amount of blood in the airway which was suctioned and evacuated. A size 6.0 endotracheal tube was placed via the bronchoscope accomplishing a selective left main-stem intubation. A left-sided chest tube was placed by the surgeon for the hemopneumothorax. A radial arterial line was placed in addition to 2 large bore peripheral intravenous catheters. The patient was placed in left lateral decubitus position. General anesthesia was maintained using sevoflurane with an end tidal concentration of 2–2.5%. Blood pressure was maintained between systolic pressure of 90 and 120 mmHg. Selective left lung ventilation was undertaken. Ventilator parameters included pressure control ventilation with tidal volume 400–500 mL, a respiratory rate between 12 and 16 cycles/min, peak end-expiratory pressure of 5 cm H_2_O, and inspired O_2_ 100% which maintained an oxygen saturation of 94–99%. A nasogastric tube was passed and esophageal injury was ruled out after no leak was detected upon injecting methylene blue through the nasogastric tube.

**Figure 4:**
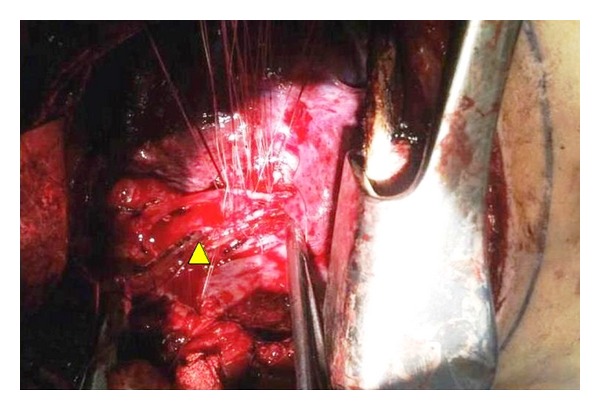
Tracheal repair with sutures along its length while the cross field endobronchial tube is in place.

The tracheal tear was repaired along its length, except at the carina where the endotracheal tube cuff was present. To accomplish this repair, the oral endotracheal tube was withdrawn and the left main-stem bronchus was intubated via the open-chest thoracotomy site with a size 6.0 sterile endotracheal tube; a sterile anesthesia circuit was used to provide cross field ventilation. The carina was then repaired. After all the sutures were in place (Figure 4) and the trachea was repaired along its full length, the cross field tube was removed and the oral endotracheal tube was advanced from the oral cavity and secured in place to ventilate both the lungs. A positive pressure breath was held to help detect any air leaks in the sutured trachea. Repeat fiberoptic bronchoscopy was performed and the airway was cleared of blood, mucus, and secretions, indicating that the back wall of the trachea appeared intact.

After the surgeons finished closing the thoracotomy, with the patient in left lateral decubitus position, a thoracic epidural was placed and dosed with 100 mcg fentanyl for postoperative pain control. Paralysis reversal was achieved using glycopyrrolate and neostigmine. The patient was then extubated while deeply anesthetized to prevent coughing. Once in recovery with the patient awake and no evident neurological deficit, an epidural test dose was given to rule out intravascular or intrathecal placement. The epidural was then bolused with 0.25% bupivacaine to achieve an appropriate level of anesthesia and patient controlled epidural analgesia (PCEA) was initiated. Postoperative recovery was uneventful. The patient was discharged home on fourth postoperative day.

## 4. Discussion

### 4.1. Etiology of Tracheal Injuries

Injury to the trachea can be extra- or intrathoracic with complete or partial disruption. Etiology of tracheal injuries includes iatrogenic causes (e.g., traumatic endotracheal intubation, the use of tube exchange catheters, percutaneous dilatational tracheostomy, and cricothyroidotomy), blunt trauma which frequently involves injuries of the trachea within 2 cm of the carina, penetrating trauma, usually to the cervical trachea, and mucosal tears, which are usually self-limiting.

### 4.2. Mechanism of Tracheal Injury in Blunt Chest Trauma

Blunt trauma usually involves the membranous portion of the intrathoracic trachea. In motor vehicle accidents, it may occur due to impact of the steering wheel or dashboard on the chest [6]. Three patters of injuries have been described [7]. In the first, rapid compression of the anteroposterior diameter of the thorax with a simultaneous widening of the transverse diameter of the chest produces lateral motion, causing traction on the trachea at the carina. Secondly, rapid deceleration causes shearing forces at cricoid cartilage and carina along the trachea, leading to their disruption. And thirdly a sudden increase in the airway pressure with a closed glottis at the time of rapid deceleration shears the bronchus from its points of fixation near the cricoids and carina. In blunt chest trauma, tracheobronchial injuries occur within 2.5 cm of the carina in 40–80% of the times [8–10] and the most common injury associated with penetrating tracheal injuries is esophageal perforation [11, 12].

### 4.3. Clinical Presentation of Tracheobronchial Injuries

If the patient is hemodynamically unstable, it is important to stabilize the airway prior to diagnosis [6]. Such injuries may also present in a subtle way. The most common presentations are dyspnea and subcutaneous emphysema [13, 14]. Other symptoms may include hoarseness, signs of external trauma, stridor, pneumothorax, pneumomediastinum or pneumopericardium, oxygen desaturation, cyanosis, and hemoptysis [15]. Extra-anatomic air is present in approximately 90% at the time of chest radiography [6]. Suspicion should be high when pneumomediastinum and pneumothorax are refractory to adequate pleural drainage [16]. In a stable patient with a chest radiograph suggestive of TBI, prompt bronchoscopy should be performed in the anticipation of surgical exploration and to confirm the location and extent of injury [13]. Computed tomographic scans may also provide additional information regarding the severity of the injury [17], such as in the cases described here.

### 4.4. Airway and Ventilation

These patients require careful airway management. A partially disrupted trachea may become completely disrupted by the passage of an endotracheal tube. There are several possible ways of airway management in these cases: (1) induction using short acting neuromuscular blocker followed by direct laryngoscopy and intubation with a double-lumen tube, (2) using short acting neuromuscular blocker followed by fiberoptic endobronchial intubation using a single-lumen tube, (3) awake fiberoptic endobronchial intubation using a single-lumen tube, (4) using a jet ventilator, and (5) cardiopulmonary bypass (CPB).

The mainstay of intraoperative management, as described in our cases, remained a single-lumen endotracheal tube [18]. An endobronchial tube may be chosen instead of a standard double-lumen tube because the double-lumen tube is usually too bulky to permit tracheal surgery [19]. Cricoid pressure can dislocate a fractured cricoid or thyroid cartilage and should thus be avoided [19]. The safest course is to maintain spontaneous ventilation, either with deep inhalation agents or topical anesthesia followed by fiberoptic intubation [19]. Tracheostomy cannot be performed in these cases due to injury in the distal airway.

After securing the airway, ventilation can be challenging due to one-lung ventilation and loss of ventilation to the atmosphere from the tracheal defect. Cross field ventilation may be set up (as described in the next section).

Other methods of ventilation such as with low-frequency or high-frequency jet ventilation and CPB have been reported earlier [20–22].

### 4.5. Cross Field Ventilation

**Figure 5:**
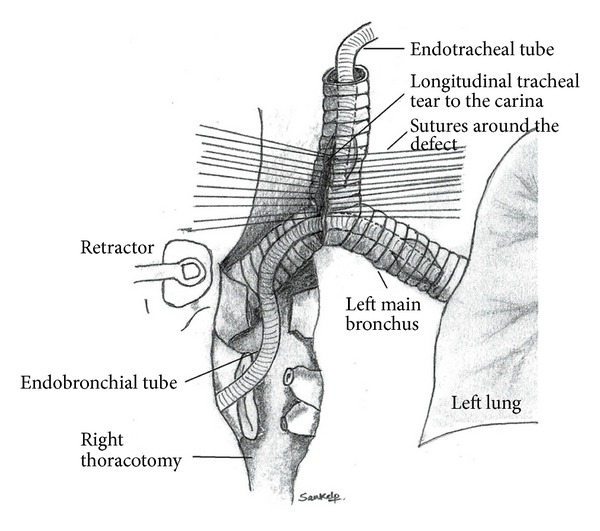
A sketch depicting a tracheal defect involving carina, the two main-stem bronchi, a retracted oral endotracheal tube, and an endobronchial tube placed via the open-chest thoracotomy to ventilate the left lung.

For ventilation during tracheal reconstruction around the carina, cross field ventilation may be required, especially if the defect involves the carina. This technique has also been described in the surgical literature as being used in complex tracheal surgeries in which a part of the trachea has to be resected due to tumor or malignancy. It involves retracting the oral endobronchial tube and intubating the bronchus across the operative field (Figure 5). A sterile anesthesia circuit attached to an endotracheal tube at its end is passed through/around the drapes across the surgical field to be placed and secured into the main bronchus for ventilating the dependent lung. Usually a small sized tube like size 6.0 or 6.5 is used. Withdrawing the existing oral endotracheal tube proximally allows the cross field tube to ventilate. This setup allows ventilation while facilitating the process for the surgeon to repair the defect around the carina. Once the anastamotic sutures are in place to repair the tracheal defect at the carina, the oral tube is advanced back into the bronchus while withdrawing the cross field endotracheal tube and the repair is then completed by tying the sutures.

### 4.6. Monitoring during Anesthesia for Tracheal Injuries

Monitoring required for tracheal surgeries, besides standard ASA monitors, includes invasive blood pressure and arterial blood gases monitoring. Oxygenation is confirmed by pulse oximetry and blood gases. An arterial catheter is also helpful in the postoperative period. It is possible to surgically compress the innominate artery that crosses the trachea at the sternal notch which may impair blood flow to the right arm and right carotid vessels. Either an arterial line or pulse oximeter on the right arm provides warning as to its occurrence [19]. A central venous catheter may be indicated for administering fluids or blood products rapidly or vasopressors and inotropes.

There are several reasons to prefer extubation after repair of such injuries. Leaving the endotracheal tube in place will irritate the tracheal anastamosis, especially if the end of the tube or cuff is at the suture line of the repaired defect. In addition, continuing positive pressure ventilation will place additional strain on the suture line that will tend to push air into the tissues [19]. Extubating the patient while deeply anesthetized may prevent disruption of the fresh repair from coughing. Constant communication between the anesthesiologist and the thoracic surgeon is imperative.

### 4.7. Postoperative Pain Control

For pain control in such injuries, while systemic narcotics remain the most prevalent modality, compared to epidural such patients retain more CO_2_, have lower PaO_2_, do not improve maximum inspiratory pressure or tidal volume, and have more respiratory and cough suppression and increased sedation. Intrapleural anesthesia, either via indwelling thoracostomy tube or placement of dedicated intrapleural catheter, provides unilateral pain control and risks pneumothorax, and compared to epidural analgesia it provides less pain relief and more narcotic use. Intercostal nerve blocks, although effective, carry high risk of pneumothorax and local anesthetic toxicity. Paravertebral blocks are unilateral, are simple to perform, improve pain, blood gases with less hypotension and urinary retention than epidural analgesia. Epidural carries procedure related complications but improves lung function and may decrease risk of pneumonia and shorten the duration of ICU stay. Postoperative pain control is improved and there is a likely decreased length of hospital stay with epidural [23] as seen in our second case.
